# Structure of the cohesin loader Scc2

**DOI:** 10.1038/ncomms13952

**Published:** 2017-01-06

**Authors:** William C. H. Chao, Yasuto Murayama, Sofía Muñoz, Andrew W. Jones, Benjamin O. Wade, Andrew G. Purkiss, Xiao-Wen Hu, Aaron Borg, Ambrosius P. Snijders, Frank Uhlmann, Martin R. Singleton

**Affiliations:** 1 Structural Biology of Chromosome Segregation Laboratory, The Francis Crick Institute, 1 Midland Road, London NW1 1AT, UK; 2 Chromosome Segregation Laboratory, The Francis Crick Institute, 1 Midland Road, London NW1 1AT, UK; 3 Tokyo Institute of Technology, Tokyo 152-8550, Japan; 4 Protein Analysis and Proteomics Platform, The Francis Crick Institute, 1 Midland Road, London NW1 1AT, UK

## Abstract

The functions of cohesin are central to genome integrity, chromosome organization and transcription regulation through its prevention of premature sister-chromatid separation and the formation of DNA loops. The loading of cohesin onto chromatin depends on the Scc2–Scc4 complex; however, little is known about how it stimulates the cohesion-loading activity. Here we determine the large ‘hook' structure of Scc2 responsible for catalysing cohesin loading. We identify key Scc2 surfaces that are crucial for cohesin loading *in vivo*. With the aid of previously determined structures and homology modelling, we derive a pseudo-atomic structure of the full-length Scc2–Scc4 complex. Finally, using recombinantly purified Scc2–Scc4 and cohesin, we performed crosslinking mass spectrometry and interaction assays that suggest Scc2–Scc4 uses its modular structure to make multiple contacts with cohesin.

## Abstract (web summary)

The cohesin complex maintains genome integrity by ensuring correct sister-chromatid segregation during mitosis and meiosis. Here, Chao *et al*. present a pseudo-atomic model of the full-length Scc2–Scc4 cohesin loader complex and reveal key Scc2 surfaces crucial for cohesin loading.

The cohesin complex (Smc1, Smc3, Scc1 and Scc3) safeguards genome integrity by ensuring correct sister-chromatid segregation during mitosis and meiosis^1^,^2^,^3^. The topological entrapment of chromosomes by cohesin is catalysed by the cohesin–loader complex Scc2–Scc4 (refs 4, 5). In yeast, Scc2–Scc4 coordinates with the remodels structure of chromatin (RSC) chromatin-remodelling complex to load cohesin at nucleosome-free regions, while mutations of the Scc4-conserved surface disrupt centromeric loading of cohesin^6^,^7^. Cohesin alone can bind DNA *in vitro*; however, the presence of the Scc2–Scc4 complex substantially accelerates its topological loading onto circular DNA in an ATP-dependent manner^5^. Functional studies have revealed that the *in-vitro*-loading activity of Scc2–Scc4 resides in the C terminus of Scc2, in contrast, Scc4 is likely a chromatin adaptor for targeting cohesin *in vivo*^7^,^8^. A number of Scc2–Scc4 contacts have been mapped on different domains of cohesin subunits that are spatially separated^5^. This has led to the suggestion that cohesin undergoes a large conformational change during loading^8^,^9^. Both rotary shadowed electron microscopy (EM) and atomic force microscopy studies have shown considerable flexibility in the Smc-coiled coils that could allow such a change to occur^10^,^11^,^12^,^13^.

Apart from sister-chromatid cohesion, cohesin also plays important roles in organizing chromosome structure and regulating transcription^14^. Cohesin interacts with Nipbl^Scc2^ and the mediator complex to connect enhancers and promoters of actively transcribing genes^15^. Chromosome conformation capture assays show that cohesin helps organize key cell identity genes into insulated neighbourhoods by the formation of chromosomal loop structures^16^, while in zebrafish and mice, Nipbl^Scc2^ and mediator cooperatively regulate gene expression during limb development^17^. The intimate relation between cohesin and Nipbl^Scc2^ manifests its importance in Cornelia de Lange syndrome (CdLS), one of a family of genetic disorders known as cohesinopathies^18^,^19^,^20^. Symptoms of CdLS include facial dysmorphism, intellectual disability and abnormal limb development, with 60% of CdLS patients carrying heterozygous mutations in the Nipbl^Scc2^ protein. Cells with one functional copy of Nipbl^Scc2^ have normal chromosome number and are not defective in sister-chromatid cohesion^21^. The disease is thought to be a result of abnormal gene expression, predominantly driven by loss-of-function mutations in Nipbl^Scc2^.

To gain further insight into the molecular function of the cohesion–loader Scc2–Scc4, which has such broad implications for both chromosome biology and genetic diseases, we determined the large modular structure of C-terminal Scc2 and derived a pseudo-atomic structure of the full-length Scc2–Scc4 complex. Our functional assays reveal key Scc2 surfaces that are crucial for cohesin loading *in vivo*. By using crosslinking mass spectrometry (XL-MS), we suggest that Scc2–Scc4 utilizes its modular structure to make multivalent contact with cohesin.

## Results

### Modular structure of C-terminal Scc2

**Figure 1:**
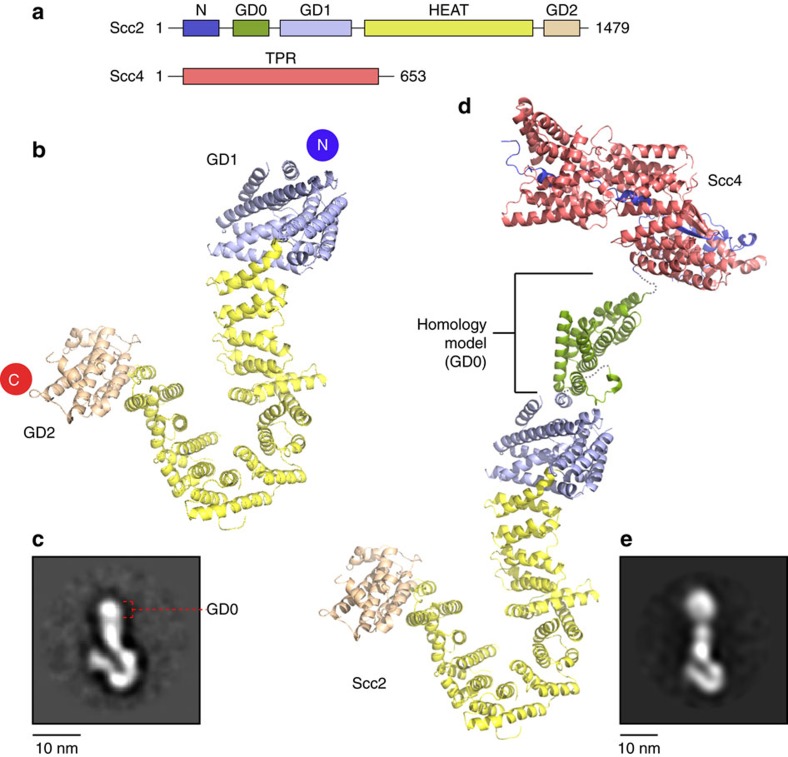
Structure of Scc2 hook and the full-length Scc2–Scc4 model. (**a**) Schematics showing the linear domain organizations of Scc2 and Scc4. The same colouring scheme is used in the corresponding crystal structures of Scc2 and Scc4 as shown in (**b**,**d**). (**b**) The modular structure of Scc2 resembles a ‘hook'. The overall fold comprises an N-terminal globular domain (GD1; violet), 14 contiguous HEAT repeats (yellow) and an oval-shaped C-terminal globular domain (GD2; wheat) with an extended loop connecting the C-terminal capping helix. (**c**) An EM 2D class average of Scc2 hook with GD0 indicated. (**d**) Reconstruction of a pseudo-full-length structure of Scc2–Scc4 with the previously determined crystal structure of Scc2^1–168^–Scc4^34–620^ (Scc2N–Scc4, PDB I.D. 5C6G; purple and salmon), a homology fold of Scc2^169–377^ (GD0; mauve) from human symplekin (PDB I.D. 3O2T) and Scc2 hook (violet, yellow and wheat). (**e**) An EM 2D class average of full-length Scc2–Scc4.

The 130 kDa structure of *Ashyba gossypii* Scc2^378–1,479^ was determined experimentally to 2.9 Å resolution by multiple isomorphous replacement with anomalous scattering (Supplementary Table 1). The Scc2 structure consists of an N-terminal globular domain (GD1) connected to 14 contiguous HEAT repeats forming an elongated structure resembling a ‘hook' (Fig. 1a,b). The 15th and 16th repeats form part of an oval-shape globular domain (GD2) with an extended loop folded back to the domain body connecting the capping helix at the extreme C terminus. The crystal structure of Scc2 hook bears good resemblance to the corresponding 2D class averages from negative-stain EM^8^ (Fig. 1c). A small domain (residues 169–377, GD0), visible in the EM classes was not present in our crystallized construct (Fig. 1a,c). Sequence analysis and homology fold prediction suggest this missing domain is predominantly α-helical, and has a tertiary fold related to that of human symplekin^22^ (Supplementary Fig. 1a). When the Scc2 hook structure is combined with the previously determined tetratricopeptide repeat (TPR) structure of Scc2^1–168^–Scc4^34–620^ (Scc2N–Scc4) and the homology fold of GD0, a model of the full-length Scc2–Scc4 complex, which resembles the EM class averages, can be derived (Fig. 1a,d,e).

Our previous EM studies showed that the hook structure of Scc2 can adopt either open or closed conformations^8^. Analysis of the atomic structure shows there are a number of loops between adjacent HEAT repeats with high crystallographic temperature factors. In addition, normal mode analyses of the structure suggest a pincer-like opening and closing of the HEAT repeats around these loops (Supplementary Fig. 1b). These loops may permit considerable motion of the hook structure as suggested by the conformational variability observed in the Scc2 hook EM images^8^. In addition, the structure of the Scc2 hook contains a number of conserved buried residues that are mutated in CdLS^18^,^20^,^23^,^24^,^25^,^26^,^27^. These mutations result in significant changes in their side-chain chemical properties (Supplementary Fig. 1c; Supplementary Fig. 2). Patients carrying these mutations show severe phenotypes, suggesting that the disruption of Scc2 structural integrity can be a causal factor.

### Surface analysis of Scc2

**Figure 2:**
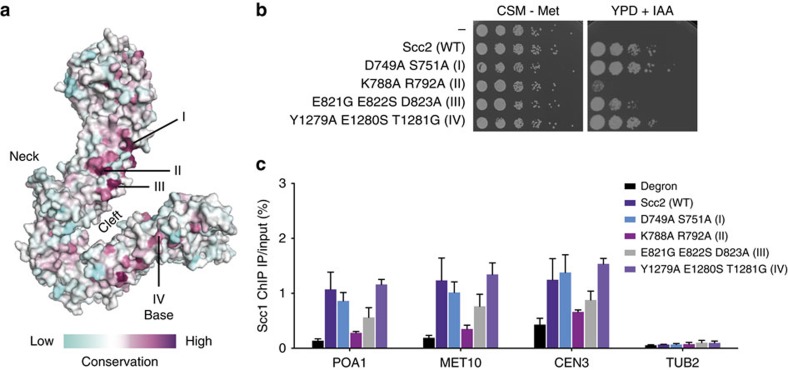
Scc2 surface analysis (**a**) Scc2 surface conservation with the positions of mutations indicated. (**b**) *SCC2* mutants were tested for the ability to restore viability to a strain carrying an *SCC2* degron allele. Cells of each strain were serially diluted and spotted on synthetic minimal medium without methionine (CSM–Met) plates or in plates containing indoleacetic acid (IAA). (**c**) Cohesin levels detected by chromatin immunoprecipitation against its Scc1 subunit followed by quantitative PCR at three cohesin-binding loci (*POA1*, *MET10* and *CEN3*) and one negative control locus (*TUB2*) in either WT or *SCC2* mutant strains arrested in mitosis. Error bars represent s.e.'s of assays each with three repeats.

Sequence alignment and conservation analysis show that the Scc2 surface is relatively poorly conserved protein with only two highly conserved patches at the neck and base regions (Fig. 2a). To investigate the importance of these conserved surfaces, we designed three sets of conserved neck surface mutations D749A/S751A (Group I), K788A/R792A (Group II) and E821G/E822S/D823A (Group III), and one set of conserved base mutations Y1279A/E1280S/T1281G (Group IV) in *Saccharomyces cerevisiae* (Supplementary Table 2). Mutant yeast strains were subjected to viability, as well as *in vivo* chromatin-binding assays (Fig. 2b,c). Our results show that both Group I and Group IV have wild-type (WT) phenotype. However, the neck mutants Groups II and III reduce cell viability, and result in cohesin-binding defects at three known cohesin chromosome-binding sites (*POA1*, *MET10* and *CEN3*).

**Figure 3:**
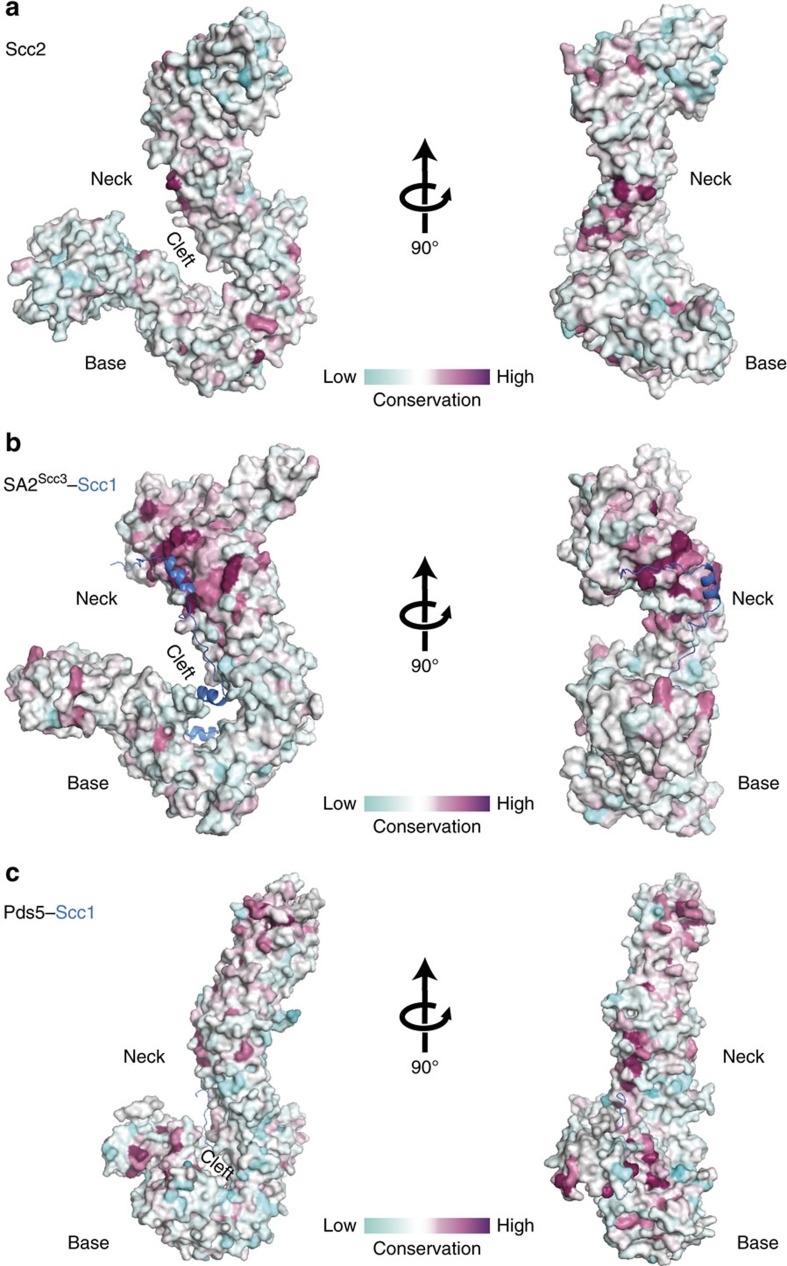
Surface conservation comparison of cohesin HEAT repeat subunits. The Scc2, SA2^Scc3^ (PDB I.D. 4PK7) and Pds5 (PDB I.D. 5F0O) structures all exhibit a hook-like curve built with strong conservation at their respective neck regions. All three structures can be presented as three regions: neck, cleftand base. (**a**) Surface conservation of Scc2 hook showing invariant (ruby) to less conserved (cyan) surfaces. (**b**) Surface conservation of SA2^Scc3^ in the SA2^Scc3^–Scc1 complex showing invariant (ruby) to less conserved (cyan) SA2^Scc3^ surfaces. (**c**) Surface conservation of Pds5 in the Pds5–Scc1 complex showing invariant (ruby) to less conserved (cyan) Pds5 surfaces.

Scc2 shares strong structural similarity with the human SA2^Scc3^ and Pds5 (refs 28, 29, 30; Fig. 3), with all three proteins exhibiting a hook-like curve built from HEAT repeats. Interestingly, the respective HEAT-repeat neck region of each protein is conserved between species, with SA2^Scc3^ utilizing its neck region to interact with Scc1 (ref. 31). Scc2 may also adopt a similar mode of interaction with its binding partners by utilizing its equivalent conserved neck surface. Due to the low affinity between Scc2–Scc4 and cohesin^5^,^6^, we employed an interaction assay using glycerol gradient centrifugation and were able to form a stable complex between recombinant cohesin and WT Scc2–Scc4. We further performed interaction assays between cohesin and Scc2–Scc4 Groups II and III mutants to investigate the significance of the Scc2 neck region in cohesin interaction (Supplementary Fig. 3a). To our surprise, neither neck mutants, which result in reduced viability and cohesin-binding *in vivo* (Fig. 2b,c), had any impact on Scc2–Scc4 interaction with cohesin as assessed by co-sedimentation *in vitro* (Supplementary Fig. 3b–g) or coimmunoprecipitation *in vivo* (Supplementary Fig. 3h). This suggests that the reduction in viability and impairment of cohesin binding to chromatin *in vivo* could be due to non-productive interaction between Scc2–Scc4 mutants and cohesin or due to the disruption of Scc2 interacting with a crucial yet unidentified binding partner through its neck region.

### Interaction studies between Scc2–Scc4 and cohesin

**Figure 4:**
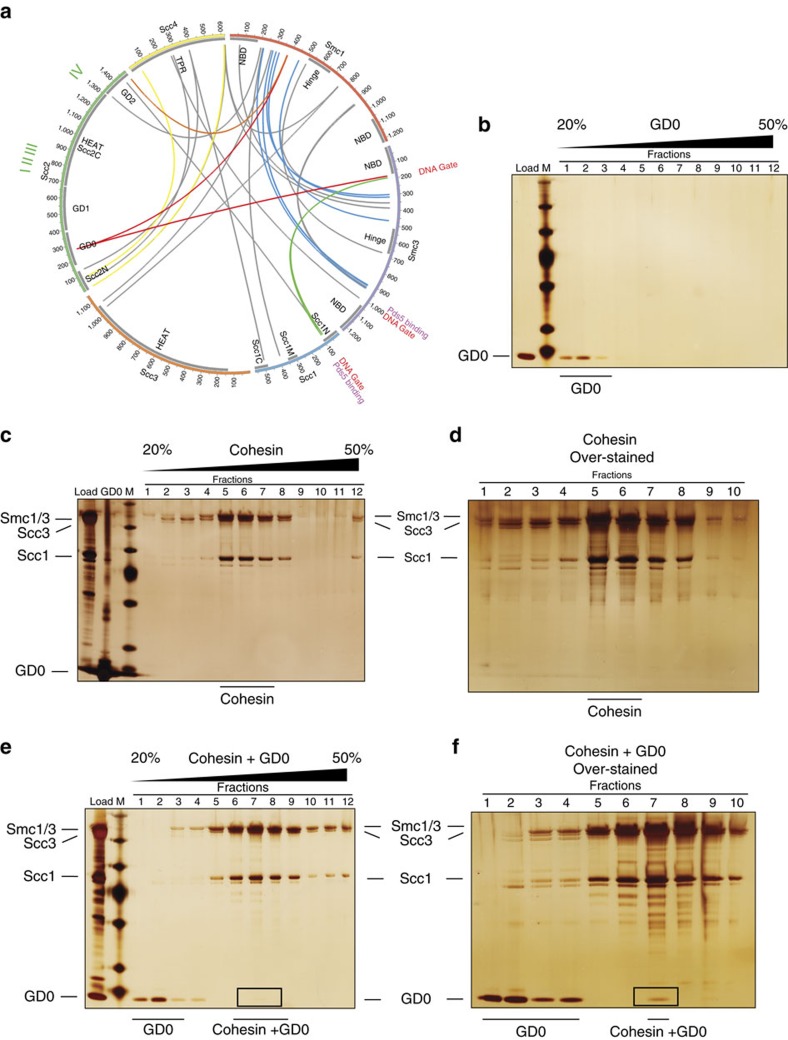
Cohesin–loader interaction analysis. (**a**) Diagram indicating intermolecular crosslinks (*n*=30) between cohesin subunits and Scc2–Scc4. Grey boxes are structured domains of cohesin and Scc2–Scc4 (Smc-coiled coils are not indicated). Red crosslinks indicate interaction between GD0 and cohesin. Crosslinks that are consistent with the published human cohesin crosslinks^12^ are in blue. Crosslinks that are consistent with published Smc3–Scc1N and Scc2N–Scc4 crystal structures^7^,^32^ are in yellow. Crosslinks that are consistent with both the published human cohesin crosslinks and crystal structures are in green. A crosslink that would require a conformation change in the loader to occur for distance restraints to be satisfied is in orange. Positions of Scc2 mutations I–IV, DNA-entry/exit gate^9^ and the documented Pds5-binding sites^12^,^28^,^29^,^38^ are marked. (**b**) Silver-stain gel showing glycerol gradient fractions of GD0 domain. M indicates protein marker. (**c**) Silver-stain gel showing glycerol gradient fractions of cohesin. (**d**) Over-stained silver-stain gel showing glycerol gradient fractions of cohesin. (**e**) Silver-stain gel showing glycerol gradient fractions of *S. cerevisiae* cohesin plus GD0 domain. (**f**) Over-stained silver-stain gel showing glycerol gradient fractions of cohesin plus GD0 with cohesin-bound GD0 being more visible.

To map interactions between cohesin and Scc2–Scc4, we performed amine XL-MS using recombinantly purified *S. cerevisiae* cohesin and WT Scc2–Scc4 (Fig. 4a; Supplementary Fig. 4; Supplementary Data 1). The inter- and intra-protein crosslinks observed are generally consistent with a similar study performed with full-length human cohesin^12^, as well as known crystal structures of Smc3–Scc1N and Scc2N–Scc4^7^,^32^, with the corresponding crosslinks highlighted in the interaction diagram (Supplementary Fig. 4a; Supplementary Data 1). The cohesin–loader crosslinks show that Scc2–Scc4 utilizes its modular structure (Fig. 1a,d) to create multiple contacts with cohesin core subunits, notably between GD0/GD2 domains and the base of the Smc1/Smc3-coiled coils. To better validate these interactions, we purified the GD0 domain in isolation (isolated GD2 could not be expressed) and tested its interaction with cohesin by glycerol gradient centrifugation (Fig. 4b-f). We were able to observe co-migration of the GD0 domain with cohesin, supporting the multivalent interactions suggested by our crosslink data. Given the high flexibility of both cohesin and the loader^8^,^12^,^33^, some caution is required in the interpretation of the crosslinks as different transient conformations of the complex might be trapped. This would explain the adjacent sites on Smc1 (residues 377 and 379) crosslinked to GD0 (residue 269) and GD2 (residue 1,491) respectively. In addition, there are also intra-molecular crosslinks within Scc2 that are consistent with the flexible conformations exhibited by the motion of the Scc2 hook and the Scc2N–Scc4 region observed in Scc2–Scc4 complex under EM^8^ (Supplementary Fig. 4a; Supplementary Data 1).

We also attempted to visualize the cohesin–loader interaction by negative-stain EM for *Saccharomyces pombe* cohesin and the cohesin–loader complex (Supplementary Fig. 5). Cohesin and cohesin–loader complexes were purified by glycerol gradient centrifugation (Supplementary Fig. 5a). Complexes could be purified in the absence of crosslinker, but more homogenous particles were observed when using mild crosslinking (Grafix) treatment^34^. When visualized, both apo-cohesin and the cohesin–loader complex adopt a rod-like conformation with a head and an extended tail that is consistent with the human cohesin recently observed under EM^33^, as well as similar structures reported in atomic force microscopy and EM studies of cohesin and condensin^10^,^11^,^13^,^35^,^36^. The heterogeneity and limited resolution of the particles does not allow detailed analysis of the interaction, but particles with a bulkier head can be readily distinguished when the loader is bound (compare Supplementary Fig. 5b,c). We propose that this represents the loader binding at or near ATPase domains of cohesin, which is consistent with our crosslinking data (Fig. 4a), and known binding sites for both Pds5 and Scc3 (refs 12, 28, 29, 31, 33).

## Discussion

We present here the large HEAT-repeat structure of the cohesion–loader Scc2 and reconstruct a pseudo-atomic structure of the modular Scc2–Scc4 complex. Our viability assays and *in vivo* chromatin-binding results indicate that the conserved surfaces at the Scc2 neck region are important for cohesin binding to chromatin (Fig. 2). However, the *in vitro* interaction assay and *in vivo* coimmunoprecipitation assay suggest that the mutations at the neck region do not impair Scc2–Scc4 interaction with cohesin (Supplementary Fig. 3). Given the equivalent region in SA2^Scc3^ and Pds5 are highly conserved and is important in protein–protein interaction in SA2(Scc3)^31^ (Fig. 3), the neck region of Scc2 may interact with a yet unidentified factor that is important for cohesin loading *in vivo*. This factor could be a chromatin remodeller, which is proposed to interact with the loader and required to clear nucleosomes away for effective cohesin loading^6^ or a chromatin target, which defines the loading site of cohesin^7^,^8^. Alternatively, the neck mutants may result in non-productive cohesin–loader interactions, which penalize cohesin loading and yeast viability. During revision of our manuscript, the C-terminal structure of the *Chaetomium thermophilum* (Ct) Scc2 was determined by Kikuchi *et al*.^37^. The authors tested the binding of individual cohesin subunits to Scc2 and showed that an N-terminal unstructured region of Scc1 interacted with Scc2. Several surface and buried mutations at the equivalent neck region of the CtScc2 reduced Scc1 interaction. Interestingly, a K1091E mutation at the equivalent neck region of the CtScc2 reduced Scc1 interaction by half. This mutation is equivalent to the K788A mutation within our Group II surface mutation pair (K788A/R792A), which reduced cell viability and cohesin binding to chromatin *in vivo* (Fig. 2). These findings, together with those of Kikuchi *et al*. support the notion that conserved surface mutations at the Scc2 neck region lead to non-productive cohesion-loader interaction, potentially due to impaired Scc1–Scc2 interaction.

Our XL-MS and glycerol gradient interaction assay results suggest that Scc2–Scc4 can form multiple contacts with the cohesin core subunits, notably between GD0/GD2 domains and the Smc1/Smc3-coiled coils adjacent to ATPase head and hinge regions (Fig. 4). In the Kikuchi *et al*.^37^ study, no interaction is detected between Scc2 and Smc1–Smc3 dimer or between Scc2 and Scc3 using *in vitro* translation-generated proteins. This could reflect that the binding of Scc2 to Smc1/3 is favoured by certain cohesin conformations, which may only exist in the context of the full tetrameric cohesin complex. The cohesin conformation that is required for Scc2 binding may involve an interaction between the Smc1/3 ATPase domains and between the adjacent Smc1/3 coiled coils (Fig. 4; Supplementary Fig. 5), which may only exist in the presence of Scc1 and Scc3. This mode of interaction between Scc2–Scc4 is similar to the interaction between full-length human cohesin and Pds5B (ref. 12). Interestingly, interactions are also seen between different Scc2–Scc4 modules and the DNA-entry/exit gate formed by Scc1 and Smc3 (refs 12, 32), suggesting that the loader might play a role in DNA gate opening. Since Pds5 also binds to Scc1 and Smc3 close to DNA-entry/exit gate^12^,^28^,^29^,^38^, this could explain how Pds5 inhibits *in-vitro* loading of cohesin by Scc2–Scc4 through competitive binding to the same site on cohesin^9^,^37^.

Our Scc2 structure contains a number of residues that are mutated in CdLS and are conserved between human and yeasts^18^,^20^,^23^,^24^,^25^,^26^,^27^ (Supplementary Fig. 1c; Supplementary Fig. 2). Patients carrying these mutations show classic phenotype of CdLS. In GD1 and the HEAT-repeat neck region lie a few buried conserved CdLS mutations, one of which (human R1895T) is required for the recruitment of histone deacetylase 3 in CdLS patients^23^ (Supplementary Fig. 1c; Supplementary Fig. 2). Further conserved CdLS mutations are located along the base region, all of which are buried within the HEAT-repeat structure. Adjacent to CdLS mutation L1584R is a buried, conserved temperature-sensitive (*ts*) mutation (*A. gossypii* D524K, *S. cerevisiae* E534K), which compromises ncRNA biogenesis, translational fidelity, and changes gene expression pattern in yeast^1^,^39^. Although different mechanisms have been proposed, the buried nature of the *ts* and CdLS mutations and their distribution along the Scc2 HEAT-repeat neck and base suggest that destabilizing these regions by CdLS mutations would compromise Nipbl^Scc2^ functions in human in ways similar to that in yeast.

Our comparative structural analysis shows that cohesin subunits Scc2, Scc3 and Pds5 are likely descendants of a common HEAT-repeat ancestor (Fig. 3), with all three proteins having been shown to bind to DNA^9^. In condensin, there are two core HEAT-repeat subunits, Ycg1 and Ycs4, both of which are required for efficient DNA binding^40^. It is possible that cohesin has evolved with two detached regulatory HEAT subunit, Scc2 and Pds5, and retained a core HEAT subunit, Scc3, whereas condensin retained both HEAT subunits as core. While yeast condensin is constitutively present on chromatin^41^, a separate loader may offer more fine-tuned regulation of cohesin loading.

## Methods

### Cloning and purification of Scc2 and Scc2–Scc4

*A. gossypii* Scc2^378–1,479^ and *S. cerevisiae* Scc2–Scc4 were amplified by PCR from genomic DNA (LGC Standards) and cloned into a modified version of the MultiBac vector pFBDM^42^,^43^. A double Strep-tag II (ds) together with a tobacco etch virus cleavage site were introduced into the C terminus of Scc2. The resultant protein expression cassettes were recombined with the DH10MultiBac cells to create a bacmid. Both constructs were expressed using the baculovirus and insect cell (High 5 cells) systems, and purified by a combination of Strep-Tactin (Qiagen), anion exchange chromatography on Poros HQ50 and Superdex 200 size-exclusion chromatography (GE Healthcare) in a final buffer of 10 mM imidazole pH 7, 300 mM NaCl and 0.5 mM tris (2-carboxyethyl) phosphine (TCEP). Mutants of *A. gossypii* Scc2 and *S. cerevisiae* Scc2–Scc4 were generated using a USER cloning method^43^.

### Crystallization and heavy-atom derivatization

*A. gossypii* Scc2^378–1,479^ in size-exclusion buffer 10 mM imidazole pH 7, 300 mM NaCl and 0.5 mM TCEP was concentrated to 6 mg ml^−1^. Crystals were grown at 4 °C in a hanging-drop manner by seeding protein solution. Seeded protein solution was mixed with an equal volume of crystallization solution containing 100 mM imidazole (pH 6.8), 200 mM lithium sulfate and 4.5% polyethylene glycol 5,000 monomethyl ether (MME) at 4 °C. Crystals started to appear after 3 days and continue to grow for 2 months. Crystals were collected either as native crystals in a cryo buffer containing 18% of polyethylene glycol 500 MME or as gold derivatives with an addition of 5 mM potassium tetrachloroaurate(III) hydrate.

### Structure solution

The structure of Scc2 hook was solved by multiple isomorphous replacement with anomalous scattering using gold and selenomethionine derivatives. Native, selenomethioine and gold data were collected on beamlines IO2, IO3 and IO4 at the Diamond Light Source. The structure was solved using the AutoSHARP package^44^ and phases (overall figure of merit=0.245) were improved using iterative solvent flattening (solvent content of 0.58) by SOLOMON. An initial model was traced using phenix.autobuild^45^. Iterative rounds of rebuilding and refinement were carried out using Coot^46^ and REFMAC5 (ref. 47). The sequence was validated by referencing the 47 selenomethionine sites located by molecular replacement of the final model against single-wavelength anomalous diffraction data. Final refinement was carried out against a native data set at 2.9 Å. Full data collection and refinement statistics are given in Supplementary Table 1. A total of 95.39% of residues were in the Ramachandran favoured region, with 0.72% outliers. A stereo image of a portion of the electron density map can be seen in Supplementary Fig. 6.

### Sequence alignments and homology modelling

Multiple sequence alignments were generated using ClustalOmega^48^. Scc2^169–377^ was used to as a search sequence for homology structures using pGENTHREADER on the Phyre2 website^49^. The human symplekin structure (3O2T) was used as a homology structure in reconstituting the pseudo-full-length structure of Scc2–Scc4 (ref. 22).

### EM image processing

Class averages of the Scc2 hook domain and full-length Scc2–Scc4 complex were re-calculated using the data presented in ref. 8. The Scc2 hook data set consists of 5,221 particles and the Scc2–Scc4 complex 2,936. Reference-free class-averages were calculated using Relion^50^.

### Normal mode analysis

Normal mode analyses of the Scc2 structure were carried out using PyANM plugin in PyMOL. A 12 Å cutoff was employed for modelling of the inter-residue interactions.

### Surface conservation analysis

Surface conservation analyses were performed using the ConSurf server^51^. Alignment sequences used for Scc2 are same as in Supplementary Fig. S2.

### *In vivo* survival assay

A strain carrying an auxin-inducible degron allele of Scc2 was transformed with integrative plasmids carrying either WT *SCC2*, D749A/S751A (I), K788A/R792A (II), E821G/E822S/D823A (III) or Y1279A/E1280S/T1281G (IV) *SCC2.* Protein levels were checked by western blot. A total of 8 × 10^6^ cells of each strain were serially diluted and spotted on synthetic minimal medium without methionine (CSM—Met) plates or in YPD plates containing 88 μg/ml indoleacetic acid (IAA).

### *In vivo* chromatin-binding assay and coimmunoprecipitation

Cells from the indicated strains were grown in synthetic medium (YNB) lacking methionine to maintain endogenous Scc2 expression that was placed under control of the *MET3* promoter. α-Factor was added to arrest cells in G1, and after 1.5 h, the culture was transferred to YPD medium to repress Scc2 expression and 88 μg ml^−1^ IAA was added to initiate Scc2 degradation. After a further 2 h, cells were released from the G1 block into YPD medium containing IAA and 5 μg ml^−1^ nocodazole. Two hours after release, when the cultures were uniformly arrested in mitosis, cell extracts were prepared and chromatin immunoprecipitation was performed as cells were fixed with formaldehyde and harvested. Protein extracts were prepared and disrupted by sonication. The DNA fragments crosslinked to a HA-tagged Scc1 were enriched by immunoprecipitation with anti HA-probe F7 antibody (Santa Cruz Biotechnology). After reversal of the crosslinks, DNA both from immunoprecipitates and from total cell extracts was cleaned up and quantified using SYBR Green Master Mix (Applied Biosystems, Life Technologies) and a ViiA 7 Real-Time PCR System (Thermo Fisher Scientific). All primer sequences used are listed in Supplementary Table 3. Cell cycle synchrony was confirmed by FACS analysis of DNA content. Strains are listed in Supplementary Table 2. For immunoprecipitation, cell extracts were prepared in EBXG buffer (50 mM HEPES pH 8.0, 100 mM KCl, 2.5 mM MgCl_2_, 10% glycerol, 0.25% Triton X-100, 1 mM DTT, protease inhibitors) using glass bead breakage in a Multi Bead Shocker (Yasui Kikai). Extracts were pre-cleared, incubated with antibody and finally adsorbed to Protein A Dynabeads. Beads were washed and elution was carried out in SDS–PAGE (polyacrylamide gel electrophoresis) loading buffer.

### Reconstitution of *S. cerevisiae* cohesin

*S. cerevisiae* Smc1, Smc3, Rad21 and Scc3 were amplified by by PCR using genomic DNA as templates and cloned into a modified pFBDM vector with a double Strep-tag II (ds) and a tobacco etch virus cleavage site at the N terminus of Rad21. The resultant protein expression cassettes were recombined with the DH10MultiBac cells to create a bacmid. S*. cerevisiae* cohesin was expressed using the baculovirus and insect cell (Sf21) systems, and purified by Strep-Tactin (Qiagen), anion exchange chromatography Porous Q, and Superose 6 size-exclusion chromatography (GE Healthcare) in a final buffer of 20 mM HEPES pH 7.5, 500 mM NaCl, 2 mM MgCl_2_ and 0.5 mM TCEP.

### Interaction assays with glycerol gradient centrifugation

WT and mutant *S. cerevisiae* Scc2–Scc4 or GD0 domain (400 nM) were mixed and incubated with cohesin (100 nM) at 4 °C for 30 min in 120 μl of buffer GG (20 mM Hepes-KOH (pH 7.5), 0.5 mM TCEP, 25 mM NaCl). A measure of 100 μl of the mixture was mounted on 5 ml of 20–50% glycerol gradient in buffer GG. The protein complexes were separated by ultracentrifugation at 4 °C at 44,000 r.p.m. for 18 h (beckman, MLA-55 rotor). The samples were fractionated by 400 μl each and analysed by SDS–PAGE and silver staining.

### Crosslinking and MS

A measure of 0.4 μM of *S. cerevisiae* cohesin was mixed with 0.8 μM of full-length *S. cerevisiae* Scc2–Scc4 and 0.8 μM of a 56- bp dsDNA in a buffer containing 20 mM HEPES pH 7.5, 50 mM NaCl, 5 mM DTT, 5 mM MgCl_2_, 1 mM ATP, and 10% v/v glycerol with addition of 5–8 mM d0- and d12-labelled DSS (Creative Molecules). The reaction was incubated at 28 °C for 40 min and quenched by adding Tris 8.0 to a final concentration of 50 mM. Crosslinked proteins were run on an SDS–PAGE gel at 200 V for 90 min, eight equal-sized bands were excised from each lane of the Scc2–Scc4+cohesin samples, and digested with trypsin overnight (Supplementary Fig. 4c). A Thermo Scientific LTQ-Orbitrap Velos mass spectrometer coupled to a Dionix UltiMate 3,000 HPLC system for on-line liquid chromatographic separation was used for data acquisition. Each sample was loaded onto a 75 μm × 50 cm C_18_ column and eluted over a 1 h gradient with collision-induced dissociation (CID) selected as the activation method. Singly- and doubly charged precursor ions and precursors of unknown charge states were rejected. Full MS spectra were acquired in the orbitrap (*m*/*z* 300–2,000, 60,000 resolution, AGC target value 1 × 10^6^), MS/MS spectra of the ten most abundant precursors from the preceding MS survey scan were acquired in the linear ion trap (CID NCE 30) and selected precursors were put on a dynamic exclusion list for 20 s. Each digested gel band sample was injected once into the liquid chromatography–MS system. The consistency of crosslinks was identified by completing three complementary experiments. Thermo Xcalibur.raw files were converted into the.mzXML format and searched using the xQuest/xProphet software package^52^. Default settings were used for the search and the data were searched against a database consisting of ScMau2, ScScc2, ScScc1, ScScc3, ScSmc1 and ScSmc3 protein sequences. False discovery rates were controlled at 1% using a target-decoy approach with the decoy database consisting of the reverse protein sequences. All liquid chromatography–MS data has now been uploaded to PRIDE with the identifier PXD004692. Comprehensive circular diagrams of intermolecular and intra-molecular crosslinks were generated using Circos (‘Circos: an information aesthetic for comparative genomics.,' 2009).

### Sample preparation for EM

*S. pombe* Scc2–Scc4 (200 nM) and cohesin (80 nM) were mixed and incubated at 4 °C for 30 min in 120 μl of buffer GG (20 mM Hepes-KOH (pH 7.5), 0.5 mM TCEP, 25 mM NaCl). A measure of 100 μl of the mixture was mounted on 5 ml of 20–50% glycerol gradient in buffer GG. The protein complexes were separated by ultracentrifugation at 4 °C at 48,000 r.p.m. for 15 h (beckman, MLA-55 rotor). The samples were fractionated by 200 μl each and analysed by SDS–PAGE and silver staining. Grafix were performed as the same protocol above but containing a linear gradient of 0–0.1% glutaraldehyde. Equivalent fractions from native and Grafix conditions were used for EM analyses.

### Electron microscopy

A measure of 4 μl of cohesin and cohesin–loader complex purified from glycerol gradient centrifugation were directly applied to glow-discharged carbon-coated Quantifoil 2/2 grids and left to blot for 12–24 h in a humidified chamber. All samples were subsequently stained with 2% uranyl acetate and grids were visualized on a Tecnai G2 electron microscope operating at 120 kV. Images were recorded on a Gatan Ultrascan 2 K at × 30,000 giving a nominal sampling of 3.45 Å per pixel.

### Data availability

Coordinates and structure factors of the Scc2 protein have been deposited in the Protein Data Bank under accession code 5ME3. The data that support the findings of this study are available from the corresponding author upon request.

## Additional information

**How to cite this article**: Chao, W. C. H. *et al*. Structure of the cohesin loader Scc2. *Nat. Commun.* **8**, 13952 doi: 10.1038/ncomms13952 (2017).

**Publisher's note**: Springer Nature remains neutral with regard to jurisdictional claims in published maps and institutional affiliations.

## Supplementary Material

Supplementary Information Supplementary Figures and Supplementary Tables

Supplementary Data 1 Full list of protein-protein crosslinks identified by mass spectrometry
