# Supplementary material for: Structure of the cohesin loader Scc2
Source: Nat Commun. 2017 Jan 6;8:13952. doi: 10.1038/ncomms13952 (PMC5227109; doi:10.1038/ncomms13952)
Supplement: Supplementary Information — Supplementary Figures and Supplementary Tables [file ncomms13952-s1.pdf]

SUPPLEMENTARY TABLE 1. X-ray data collection and refinement statistics.

	Native 2	Native 1	Gold	Selenium
**Data collection**				
Space group	$P2_1$	$P2_1$	$P2_1$	$P2_1$
Cell dimensions				
a, b, c (Å)	98.05, 106.4, 143.18	100.17, 108.62, 148.46	100.13, 108.47, 148.17	100.08, 107.90, 147.77
α, β, γ (°)	90, 104.4, 90	90, 106.72, 90	90, 106.56, 90	90, 106.42, 90
Resolution (Å)	48.81 - 2.85 (2.952 - 2.85)*	142.18 - 3.3 (3.42 - 3.3)	142.4 - 3.42 (3.56 - 3.42)	107.9 - 3.50 (3.66 - 3.50)
R_{merge}	0.07152 (1.244)	0.161 (2.062)	0.174 (2.410)	0.131 (1.927)
$I / \sigma I$	10.36 (0.75)	8.2 (1.0)	8.2 (0.9)	7.6 (1.0)
Completeness (%)	0.99 (0.96)	100 (100)	100 (100)	99.6 (100)
Redundancy	3.3 (2.9)	6.8 (7.0)	6.9 (6.8)	6.9 (7.0)
**Refinement**				
Resolution (Å)	2.85 (2.8907 – 2.8500)			
No. reflections	65853 (2429)			
R_{work} / R_{free}	0.2239 (0.3715) / 0.2678 (0.4019)			
No. atoms				
Protein	16017			
Ligand / Ion	0			
Water	0			
B-factors	96.43			
Protein	96.43			
R.m.s. deviations				
Bond lengths (Å)	0.013			
Bond angles (°)	1.74			

*Highest resolution shell is shown in parenthesis.

SUPPLEMENTARY TABLE 2. Yeast strains employed in this study.

All strains were in a W303 background.

Y4820 *MAT*a *pADH1-OsTIR1-myc9::ADE2, URA3::pMET3-scc2-IAA17::kanR, SCC1-HA3::LEU2*

Y4818 *MAT*a *pADH1-OsTIR1-myc9::ADE2, URA3::pMET3-scc2-IAA17::kanR, SCC1-HA3::LEU2, HIS3::pSCC2-SCC2-Pk3*

Y4906 *MAT*a *pADH1-OsTIR1-myc9::ADE2, URA3::pMET3-scc2-IAA17::kanR, SCC1-HA3::LEU2, HIS3::pSCC2-SCC2(D749A/S751A)-Pk3*

Y4907 *MAT*a *pADH1-OsTIR1-myc9::ADE2, URA3::pMET3-scc2-IAA17::kanR, SCC1-HA3::LEU2, HIS3::pSCC2-SCC2(K788A/R792A)-Pk3*

Y4908 *MAT*a *pADH1-OsTIR1-myc9::ADE2, URA3::pMET3-scc2-IAA17::kanR, SCC1-HA3::LEU2, HIS3::pSCC2-SCC2(E821G/E822S/D823A)-Pk3*

Y4909 *MAT*a *pADH1-OsTIR1-myc9::ADE2, URA3::pMET3-scc2-IAA17::kanR, SCC1-HA3::LEU2, HIS3::pSCC2-SCC2(Y1279A/E1280S/T1281G)-Pk3*

Y4910 *MAT*a *pADH1-OsTIR1-myc9::ADE2, URA3::pMET3-scc2-IAA17::kanR, SCC1-HA3::LEU2, HIS3::pSCC2-SCC2(K1454S/L1456A/K1459S)-Pk3*

Y4911 *MAT*a *pADH1-OsTIR1-myc9::ADE2, URA3::pMET3-scc2-IAA17::kanR, SCC1-HA3::LEU2, HIS3::pSCC2-SCC2(K1329A, K1332A)-Pk3*

Y5196 *MAT*a *pADH1-OsTIR1-myc9::ADE2, URA3::pMET3-scc2-IAA17::kanR, SCC1-HA3::LEU2, HIS3::pSCC2-SCC2(R1115A, K1119A, K1123S)-Pk3*

SUPPLEMENTARY TABLE 3. Primers for real-time PCR.

POA1-F	AAACGGCCACATCAAATACC
POA1-R	TCCAAGGGACTCCGAATATG
MET10-F	ACTTGTGTGGCCCTACTTGG
MET10-R	CGACTTTGATGCCTCTTTCC
CEN3-F	CGCCACTTTAACAAATGTGC
CEN3-R	GCAGAACCACCGTAGCAGTT
TUB2-F	TTACCGTTGCAGCCTTCTTT
TUB2-R	TGAGGAGCGACAGAACACAC

a

```
Symplekin      MTTSERVVDLLNQAALITNDSKITVLKQVQELIINK-DPTLLDNFLDEIIAFQADKSIEV 88
GD0            ---------SLNRVALGS--QHLKTLERLMQYIGADESSAEFGDF-------EY--WITL 202
                         **:.** :  .::..*::: : *  . . : : :*       :    * :

Symplekin      RKFVIGFIEEACKRDIELLLKLIANLNMLLRDENVNVVKKAILTMTQLYKVALQWMVKSV 148
GD0            EDRATHILSEQCIDKLHIVFRNILSIPAVWTELDVSL----------------------- 239
               .. .  ::.* *  .:.:::: * .:  :  : :*.:

Symplekin      ISELQEACWDMV----------------------SAMAGDIILLLDSDNDGIRTHAIKFV 186
GD0            LQRIMDVCVSTITICLEKIELKEYSHDYTLIAFGSSMVVFFIFLLDQNDRRLQLEQYMMV 299
               :..: :.* . :                      *:*.  :*:***.::  :: .   :*

Symplekin      EGLIVTLSPRMADSEIPRRQEH-DISLDRIPRDHPYIQYNVLWEEGKAALEQL--LKFMV 244
GD0            PLQFLTFTADNLEEEFDRGGNGVERSLA---MLHSTLDVFYMYISNKPKLDDGLVVKLVY 356
                  ::*::    :.*: *  :  : **      *  ::   :: . *  *::   :*::

Symplekin      HISSINLTTALGSLANIARQ 264
GD0            MFTELIMSTWSHNVSSVSLQ 382
                ::.: ::*   .::.:: *
```

b

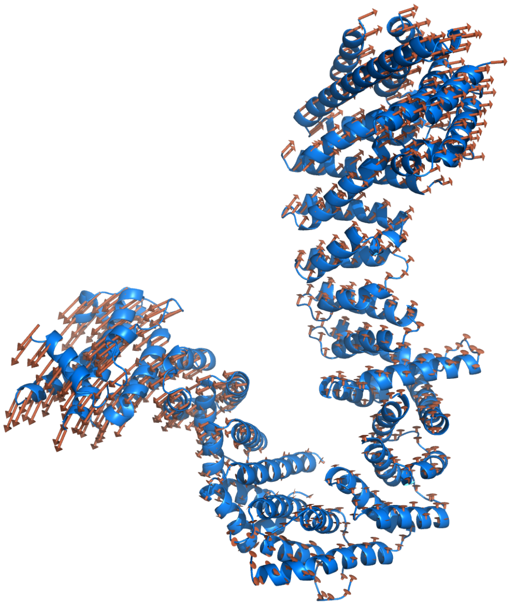

c

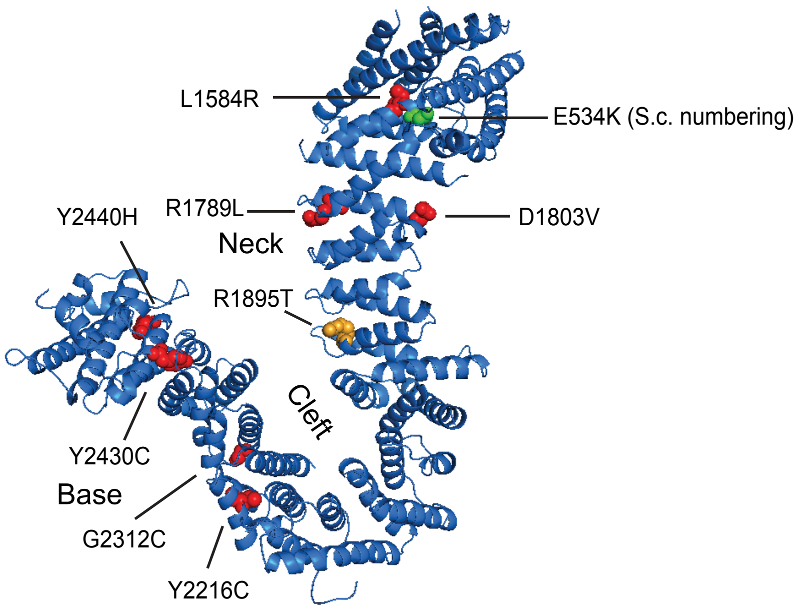

Supplementary Figure 1. GD0 alignment and CdLS mutations
(a) Sequence alignment between GD0 and human symplekin.
(b) Normal mode analyses (NMA) of Scc2 hook structure with arrows indicating major predicted normal mode motion.
(c) Scc2 hook structure with a yeast ts mutation (S. cerevisiae E534K, green) and the equivalent human CdLS mutations (red and gold) indicated. Gold mutation is responsible for the recruitment of HDAC3. The buried nature and changes in chemical properties of the CdLS mutations suggest that structural integrity of the human NipblScc2 would be compromised.

```
AgScc2          -----LEEEFDRGGNGVERSLAMLHSTLDVFYMYISNKPKLDDGLVVK--LVYMFTELIM 363
ScScc2          -----LKILTIEYG-SLKIEFDTFQEALELLPKYIRNGPFLDDNVTAK--LVYIFSDLLM 372
SpScc2          IVQSKTSDARTSEIQHLSVLLQKVANVLNILSKVAHEIP-LSEAVVIR--IVYLFPKVST 430
CtScc2          RNTSDTEELFKALSSCKRRILPLMTDAQKLLSMMSELIAKVDTSETVTNALEFAASQLLF 617
HsNipbl         ------GGLLSSKAKRAKCSTHKQRVIVMLYNKVCDIVSSLSELLEIQ----LLTDTTIL 1417
                                             :        . :.

AgScc2          STWSHNVSSVSLQN-HIGNVKTIGMKSIEVLFEKLPEQRLFIIDELLSHLDNLPNTRSQK 422
ScScc2          NNDIEATTNIQFQS-FWDNVKRISSDILVSLFGSFDQQRGFIIEELLSHIEKLPTKRIQK 431
SpScc2          LDNSFKTKLPNCNSSSFDFLKAPLFQTLQYLFRLYPYQRDFIIEESLTNFSHLPTARSVS 490
CtScc2          IETANAERDSVIETQKFDGFRLVAMDMICQIFLLNPSQRQGIIDEILTSLEKLPLGKRAR 677
HsNipbl         QVSSMGITPFFVEN--VSELQLCAIKLVTAVFSRYEKHRQLILEEIFTSLARLPTSKRSL 1475
                            :.   . .:    . :  :*      :*  *::* :: : .**  :

AgScc2          RMKNIG------KNLHITHFSYIMLSILQVWNNYDYCSNLVSPDT--------------- 416
ScScc2          KLRKVGN-----QNIYITDFTFTLMSMLENINCYSFCNQMKDIAP--------------- 471
SpScc2          RTYRLS------NGKSIQYYSTLFVRLIQSCSIQNLFDSEIVQSESKS------------ 532
CtScc2          TFKLAD-------GSSIQPVSALIMRLVQTSAGKDEGRGSSGRLDMTEGVQKVTGAKAFI 730
HsNipbl         RNFRLNSSDMDGEPMYIQMVTALVLQLIQCVVHLPSSEKDSNAEED-------------- 1521
                     .          *   :  .: :::

AgScc2          ------------EQLHDLIAHHAATQSDLEFAADHIIDTILKKCFSNI-----SKYRFVF 504
ScScc2          ------------ENIDLLKNEYKKQEEFLFNIVEHINDTILERFFKNP-----SALRYVI 514
SpScc2          --TEALHSGNLTEHLKTVESILSKSRHEEYRIANHIVAYLLSRSLKQNKTESDNSFAILT 590
CtScc2          IQSEAQGASQHSTAIRDLDTISTPLLETARNNASYVIKFIVNRALNSTKS-GDTPYRNLL 789
HsNipbl         -----------SNKKIDQDVVITNSYETAMRTAQNFLSIFLKKCGSKQGE---EDYRPLF 1567
                                              .. .   ::.:  ..           :

AgScc2          DQFVTDMTTVVTLPDWPVSDLILARLLKKLLSIFNPQSQKHIN-------IESVALQGIG 557
ScScc2          DNFVQDLLLLISSPQWPVTEKILSSLLKRLLSVYSPSMQVSAN-------IETICLQLIG 567
SpScc2          KILLEDLLNMLSLPEWCGTETIIRQFAMNLVMTVTNDKQAVSSKNAALDLISLIVNKVLA 650
CtScc2          DLFVEDFITCLDNPDWPAAELLLRLLMLMMVGLVENDKSSIMAKNMALELLGTMCAAISK 849
HsNipbl         ENFVQDLLSTVNKPEWPAAELLLSLLGRLLVHQFSNKSTEMALRVASLDYLGTVAARLRK 1627
                . :: *:   :   *:*  :: ::  :   ::      .            :  :
                 ScE534K ts mutation[1] L1584R[25]
AgScc2          LIGSTILDIRLSS------------NSNPTANLIHLFNYPEDIDG--LFKA-YRTCLAYC 602
ScScc2          NIGSTIFDIKCST------------RDHEDNNLIKMINYPETLPH--FFKS-FEECIAYN 612
SpScc2          LFDLSLFEKHNIP------------APTNFNDIISFIPSITRLNE--LSQVSFNHFYFLC 696
CtScc2          LRGHVRKMASALE------------ADELSLFLSDLAASALELKS--RPEHMVAWTGPYR 895
HsNipbl         DAVTSKMDQGSIERILKQVSGGEDEIQQLQKALLDYLDENTETDPSLVFSRKFYIAQWFR 1687
                                             :                 .

AgScc2          TNSVQHHTPYKKFLWCKQLD-------------VLTKLKEMDKESQGWGSKLQQKFLSLIE 649
ScScc2          ETIKCRRSATRFLWNLRLG-------------TILILEEYTKDAKEQIITVDNELKKILE 659
SpScc2          KGDISLENILPYNYNKWFS-------------FLLQLRKVCNDS-EALKIIDNCIDKNMQ 742
CtScc2          ATLEYLQSRSRFNEDAQLSSAMTFIISEWGSKICTCYDGYQDDVLERDQELGRLAYRLRE 955
HsNipbl         DTTLETEKAMKSQKDEESSEG-----------THHAKEIETTGQIMHRAENRKKFLRSII 1736
                      ..         .                                   .

AgScc2          SIH----LPLEQA-ANLGAVEILPTYCSTLLTSELINMYEPYLKLVLSLLERHKVKLRSG 704
ScScc2          QIKDGGLGPELEN-READFSTIKLDYFSILHAFELLNLYDPYLKLILSLLAKDKIKLRST 718
SpScc2          KSQEGFQGPSPFK-ADENDEDI---FIISLYHSSLFLNLKFFVSLIIGFLDSPSQASLRTK 798
CtScc2          MILDRKWLSTEYTYKDVSPLQAKLSYSIILLRSQFCEAFGAILNILLNSMASDQPTVRSK 1015
HsNipbl         KTTPSQFSTLKMNSDTVDYDDACLIVRYLASMRPFAQSFDIYLTQILRVLGENAIAVRTK 1796
                        .        .               :       :. ::  :       :*:
                                                                   R1789L[18]
AgScc2          AIRCLALLISKDKNMLY-TPIVKETIENRLTDSSPLVKDAILELI-----ELGSSYIDFY 758
ScScc2          AIKCLSMLASKDKVILS-NPMVKETIHRRLNDSSASVKDAILDLV-----SINSSYFEFY 772
SpScc2          CLRIINQMKTIPSILRT-HPEVLAQIISKSNDQSAIVRDTVLDLLGTYIMAYRETIPQIY 857
CtScc2          SLKSINQVMEADPTILDGDSVVVQLILRSSSDSSTQVRDSALGLISKCISLRPALEEKMT 1075
HsNipbl         AMKCLSEVVAVDPSILA-RLDMQRGVHGRLMDNSTSVREAAVELLGRFVLCRPQLAEQYY 1855
                .:: :  :      :     :   :      *.*. *::: : *:                .
                          D1803V[18]
AgScc2          QHININYNDDSMLVRKHVLRMNQIIYDDTEDIIIKAYVASRILRRIEDEEDVIIETARSE 818
ScScc2          QQINNNYNDDSIMVRKHVLRINEKMYDETNDIVTKVYVIARILMKIEDEEDNIIDMARLI 832
SpScc2          GCIISGISDPSTIVRKRAIKQLCEVYEATEDLNIRVDIASKLLTRSNDEEETISELSLEV 917
CtScc2          ETVVNRFSDAGPGVRKRAMKLAKDIYLRNSNRVLRSAIANGLLHRVQDPEESVRDLARQV 1135
HsNipbl         DMLIERILDTGISVRKRVIKILRDICIEQPTFPKITEMCVKMIRRVND-EEGIKKLVNET 1914
                  :     * .  ***:.::    :           :   :: : :* *: :  .
                                                        R1895T (HDAC3 binding)[23]
AgScc2          LLKRWILSIQCPNAKPE-----LQIKNCRVSIRVIAQLLVGGDKICDLFEHFLIFYVLNK 873
ScScc2          LLNRWILKVHEVLDQPE-----KLKEISSSVLLVMSRVAIMNEKCSQLFDLFLNFYLLNK 877
SpScc2          LEKLWFSPASNELDCQKGYEQLTFLEKQKLRVQYFPILKLCAEPSTERHVLLVTSLKTML 977
CtScc2          IEEIWFAPFHSGETSAA----------SQISLSEHVILMVQTVKRGNVANVLDKVLQALL 1185
HsNipbl         FQKLWFTPTPHNDKEAM----------TRKILNITDVVAACRDTGYDWFEQLLQNLLKSE 1964
                : : *:                             :     :        :    :

AgScc2          NAHTEDQNKLISSSLCLLTDQVIEMVIENEAADVDNQR-EEEGRNIMKFLSVLSSCQDSF 932
ScScc2          EAHSKEAYDKITHVLTILTDFLVQKIVELNSDDTNEKNSIVDKQNFLNLLAKFADSTVSF 947
SpScc2          TSKEEINLSTLHTQIRLLLSCLFNQLIEVVTEDQVDESTKGILYEIMSTLFVFSRAFPFL 1037
CtScc2          SPSSKTSQASMEVCRKLVG-----SMFDLIDNIDSNDASAPSGRDVLQVLMIFAKAEASL 1240
HsNipbl         EDSSYKPVKKACTQLVDNLVEHILKYEESLADSDNKGVNSGRLVACITTLFLFSKIRPQL 2024
                                          :       .          :  *  ::     :

AgScc2          ITKDHITALYPYLHSD---TKSDFQLHILKVYRNTFEQLSHFKPKFLYDLETTILSRLPR 989
ScScc2          LTKDHITALYPYMVSD---EKSDFHYYILQVFRCTFEKLANFKQKFLYDLETTLLSRLPK 1004
SpScc2          FDLSYLHLLKPYLRSASTIEEQRFLYYVVAIFRQVLPFQKEISESFLRSLESVLLQRLTK 1097
CtScc2          FTFEQLRLLRPYIASIGTSEDLTVSRAVVVIYRRVLPQLSSAHAQFLTDVRKELLPVVAK 1300
HsNipbl         MVKHAMTMQPYLTTKCSTQNDFMVICNVAKILELVVPLMEHPSETFLATIEEDLMKLIIK 2084
                :    :        .     .  .   :  : . ..        **  :.  ::  : :
```

```
AgScc2          MNVRELDEAIPLSWSLSRHRKDDTRICKACASCLGQLSPYIANATTD---PSAVRPDGKL 1046
ScScc2          MNVREIDEAMPLIWSVATHRHDTARVAKACSSCLSHLHPYINKANNE---EAAIVVDGKL 1061
SpScc2          AGTATLMEIVPCLCSLFTRLNDYERLKKIVVSCLKSLEEARHSENNF---QKMVR----L 1150
CtScc2          VPRALLDDVMACLWIISTLLGTYEPLARLVISSLKGIQKTRASAQVQPLDQLKIRQFDRY 1360
HsNipbl         YGMTVVQHCVSCLGAVVNKVTQNFKFVWACFNRYYGAISKLKSQHQE--DPNNTSLLTNK 2142
                     : . :.    :         .     .          .

AgScc2          QRLLYLATGFARFCSF-ENTEGKFPNLKTRENIFEYVTKCLLMFTKENIHHVIRRIATKN 1105
ScScc2          QRLIYLSTGFARFCFP-KPSNDKIAFLQEGETLYEHITKCLLVLSKDKITHVIRRVAVKN 1120
SpScc2          IDLIGLFSRYGDLNRINDDWKHSLDFISPECDDAYVILLGYFQKLLKDAKGQLRIHIIDN 1210
CtScc2          SLIVGMAGKHCNLDSHHEMFKEHFPKFS-GASVSKLMVDIVVPFAAPSWPLDVRKPALDC 1419
HsNipbl         PALLRSLFTVGALCRHFDFDLEDFKGNS--KVNIKDKVLELLMYFTKHSDEEVQTKAIIG 2200
                  ::         :    .     :   .             .          ::

AgScc2          LVKIASRYPKLFNSRHVLTVLDTQFEKGI--LDIQLVVLESLYDFFLAEE-----RRSLI 1158
ScScc2          LTKLCGNHPKLFNSRHVLHLLDKEFQSDQ--LDIKLVILESLYDLFLLEE-----RKSVR 1173
SpScc2          MSRICLRETSLFISPLMLSTLDMIIAENN--VNEVSVLFKSFLELLAADEDLIFEADQKL 1268
CtScc2          VGLVCQSSPRNYVAANVYTAFQQVFDDQI--PILETMVLRSLKEFLFSEE-------KRS 1470
HsNipbl         LGFAFIQHPSLMFEQEVKNLYNNILSDKNSSVNLKIQVLKNLQTYLQEED---------- 2250
                :       .       :    :  : .          ::..:   :  ::
                    Y2216C[24]
AgScc2          QVGVDGTISSNNELRKVVANHTKSDSINDGICSALVSRYLDKILKICLIADLNNAMVAIR 1218
ScScc2          NTGVNSTLSSNSILKKKLLKTNRVEFANDGVCSALATRFLDNILQLCLLRDLKNSLVAIR 1233
SpScc2          SLKGKQNVQSNKSVDRDMLKGTKDKQWIEGVSASLMQHFLPCILDSCFSKNLRYSMLGIE 1328
CtScc2          EQEPEGPAGKDGESKKKRELTVIGGTNYDDVASATTHRFLKDITRIATSTQDDHAFLAVE 1530
HsNipbl         --TRMQQADRDWKKVAKQEDLKEMGDVSSGMSSSIMQLYLKQVLEAFFHTQSSVRHFALN 2308
                          :                 ..:.::    :*  :       :     ..:.

AgScc2          FLKQILTYGYTNPSLCVPTVIALTASPNSYMRDLSSEMFSELLQGYESMAFNCLNQGIRL 1278
ScScc2          LLKLILKFGYTNPSHSIPTVIALFASTSQYIRHVAYELLEDLFEKYETLVFSSLSRGVTK 1293
SpScc2          ILKCIIHQGLVNPRMCFSTIIALESNAIKETREVAILLHTELHRRHESLIDGLYAQSADL 1388
CtScc2          VLASINRQGLVHPKETGVTFITLATSTHPRISELAFLEHKALHTKHETVIEREYAKAIQS 1590
HsNipbl         VIALTLNQGLIHPVQCVPYLIAMGTDPEPAMRNKADQQLVEIDKKYAGFIHMKAVAGMKM 2368
                .:      *  :*      .*:: :..     . :      :   :  .       .
                        G2312C[24]
AgScc2          GTEYAVKIRPK-QFHEDSMFLRRLQQLMSTNKRNRSKFLKVVKKTVFSNYLSTSVNYEES 1337
ScScc2          AIHYSIHTDEK-YYYKHDHFLSLLEKLCGTGKKNGPKFFKVLKR-IMQSYLDDITDLTST 1351
SpScc2          IFS-LQKTEEY-QTFKLGEFSPFQSAYTIVSADKSSKSRKKLIMQILKPLKLDGIDLPSF 1446
CtScc2          IFAYQRDIVKDPRGATTNPFTPKLHLFMEVLKISKAKNRVKFLEKLVSQIDFDIAKLDMS 1650
HsNipbl         SYQVQQAINTCLKDPVRGFRQDESSSALCSHLYSMIRGNRQHRRAFLISLLNLFDDTAKT 2428
                                 .               .  :         ..       .

AgScc2          ------CYHIIYLLHNLSKISYDNMLELYEMLRSVISVSEYFSDRISERL-DGFQSN--- 1387
ScScc2          NSS--VQKSIFVLCTNISNITFVSQYDLVSLLKTIDLTTDRLKEVIMDEIGDNVSSL--- 1406
SpScc2          TEE--KVSFVSFCCVCLAGIPYVSIEEPLMIISTVDSVLATIGPTITGWMKKLDHER--- 1501
CtScc2          EELPPHVQYSRFIIENLAFFEYVTVGEIHSLVAAMERLVASTGASVAQVIESEVFHLRID 1710
HsNipbl         DVT-----MLLYIADNLACFPYQTQEEPLFIMHHIDITLSVSGSNLLQSFKESMVKDKRK 2483
                                ::  :  : .  :   ::  :          :   :  .
                           Y2430C[20] Y2440H[18]
AgScc2          ---------------------------------------------------QNTAAEVSA 1396
ScScc2          ---------------------------------------------------SVSEEKLSG 1415
SpScc2          ---------------------------------------------------FKILAGINL 1510
CtScc2          VLDAPSSQSPNPDGQLRAEAQTQGQVNPLLPDIE-----------------YSRLRQLTA 1753
HsNipbl         ERKSSPSKENESSDSEEEVSRPRKSRKRVDSDSDSDSEDDINSVMKCLPENSAPLIEFAN 2543
                                                                         .

AgScc2          IIVTRLSIEEFKRFMFEQHHLSEAKLTLLDATDDENLRNKSVAALDDQVGSLHMENIFLG 1456
ScScc2          IILIQLSLQDLGTYLLHLYGLRDDVL-LLDIVEESELKNKQLPAKKPDISKFSAQ---LE 1471
SpScc2          CNLIYLKRYIKYAFSISDSSRPIREKKPLTLLNRGYVDLITSDAKPDIVSKLVIK--LFE 1568
CtScc2          ASMILLAIWEVRTYLRRLYSLGTNRR--ENSAKPAKPQVKDLARPPVKVQGVTGDKVWEE 1811
HsNipbl         VSQGILLLLMLKQHLKNLCGFSDSKIQKYSPSESAKVYDKAINRKTG--VHFHPKQTLDF 2601
                     *       .                  .                  .  .

AgScc2          YENPINNKDYCWKYISTLHRDEI------------------------------ 1479
ScScc2          NIEQYSSNGKLLTYFRKHVKDT------------------------------- 1493
SpScc2          EENILSGEDQVEGEQLTVV---------------------------------- 1587
CtScc2          INNIMGALSGGRERMVRACKDLVELMSIDKEFLVPSEEDEMDLDAAGEE--- 1944
HsNipbl         LRSDMANSKITEEVKRSIVKQYLDFKLLMEHLDPDEEEEEGEVSASTNARNK 2653
                  .
```

Supplementary Figure 2. Sequence alignment of Scc2 hook.

Temperature-sensitive (green) and Cornelia de Lange mutations (red and gold) are labelled. Ag – A. gossypii; Sc – S. cerevisiae; Sp – S. pombe; Ct – C. thermophilum; Hs – H. sapiens. The same alignment is used for Scc2 surface conservation analysis.

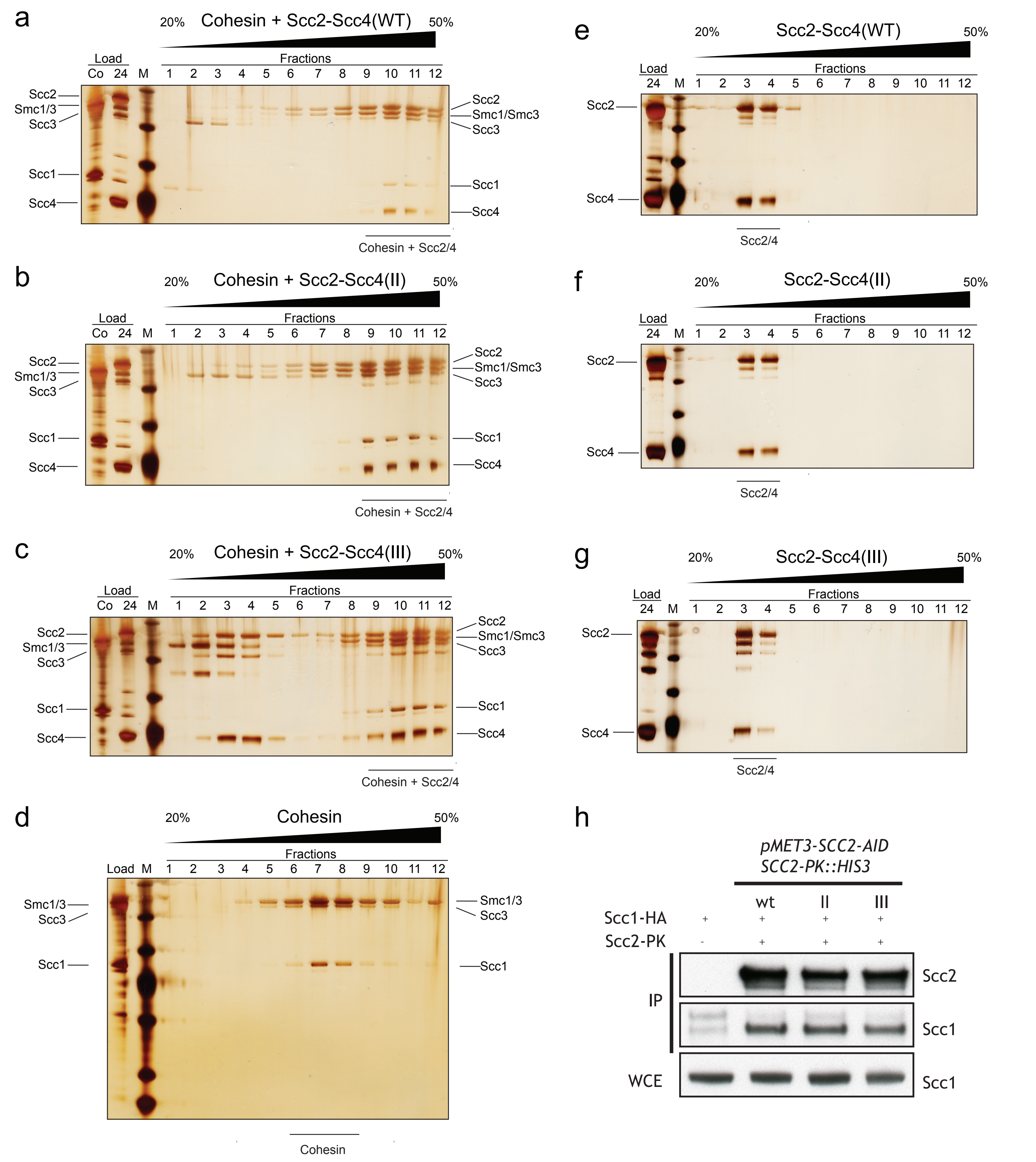

Supplementary Figure 3. Scc2-Scc4 neck mutations do not affect cohesin binding.
(a) Silver-stain gel showing glycerol gradient fractions of S. cerevisiae cohesin plus wild-type Scc2-Scc4.
(b) Silver-stain gel showing glycerol gradient fractions of S. cerevisiae cohesin plus Scc2 (K788A/R792A)-Scc4 (Group II mutant). Cohesin interacts normally with Group II Scc2-Scc4 mutant.
(c) Silver-stain gel showing glycerol gradient fractions of S. cerevisiae cohesin plus Scc2 (E821G/E822S/D823A)-Scc4 (Group III mutant). Cohesin interacts normally with Group III Scc2-Scc4 mutant.
(d) – (g) Silver-stain gel showing control glycerol gradient runs.
(h) Co-immunoprecipitation showing that Group II and Group III mutants interact with cohesin same as wild-type Scc2 in vivo.

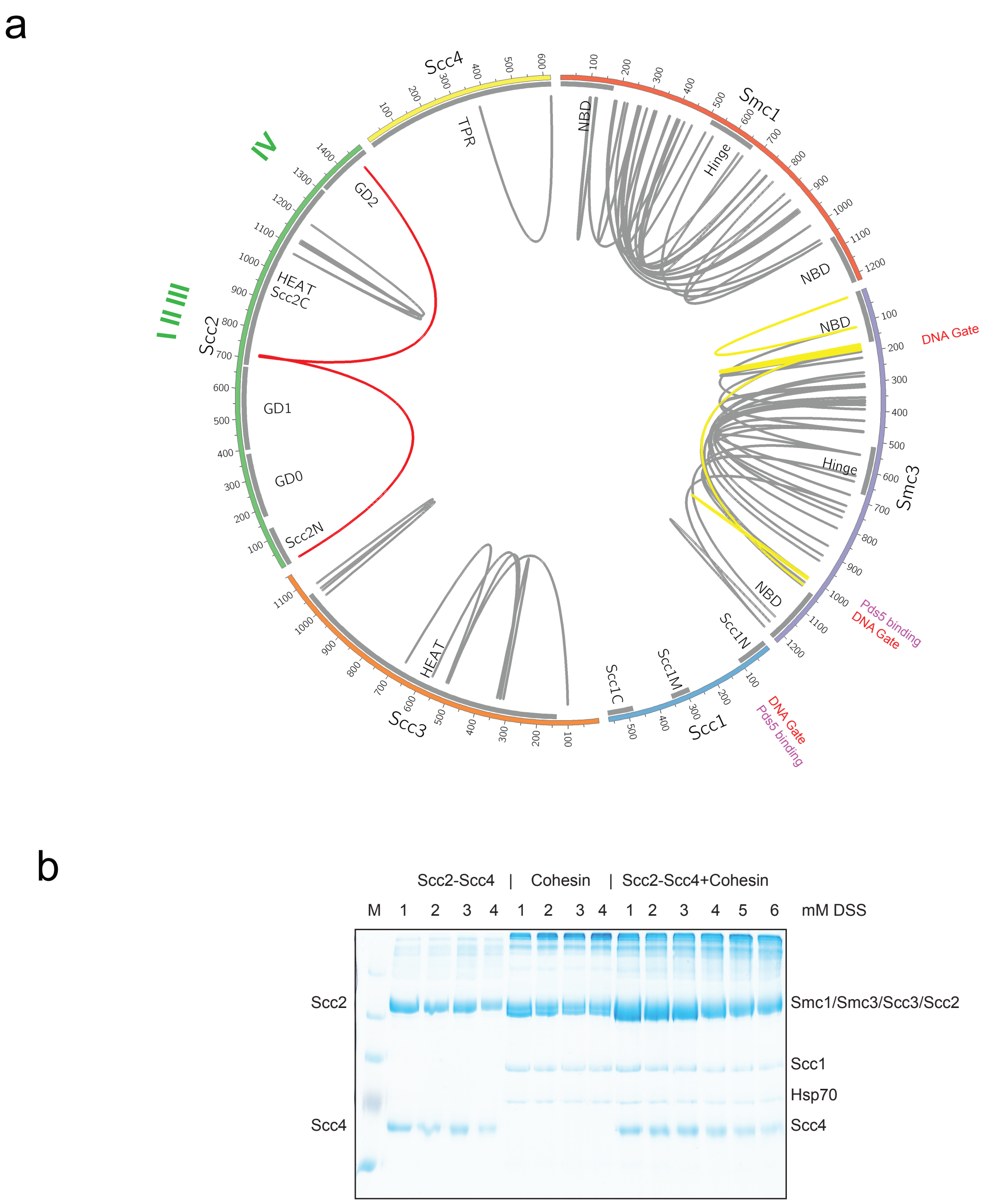

Supplementary Figure 4. Crosslinking/mass spectrometry analysis

(a) Diagram indicating intramolecular crosslinks (n = 108) within cohesin and loader subunits. Crosslinks that indicate the flexible conformations of Scc2-Scc4 seen under EM are in red. Crosslinks that are consistent with published Smc3-Scc1N and Scc2N-Scc4 crystal structures[7,32] are in yellow. All intramolecular crosslinks are further than 20 residues apart.

(b) An example of SDS-PAGE gel showing crosslinked samples between cohesin and Scc2-Scc4 used for mass spectrometry analysis. The reaction was incubated at 28 °C for 40 minutes and quenched by adding Tris 8.0 to a final concentration of 50 mM. Eight equal-sized bands were excised from each lane of the Scc2-Scc4 plus cohesin samples for trypsin digestion and mass spectrometry analysis.

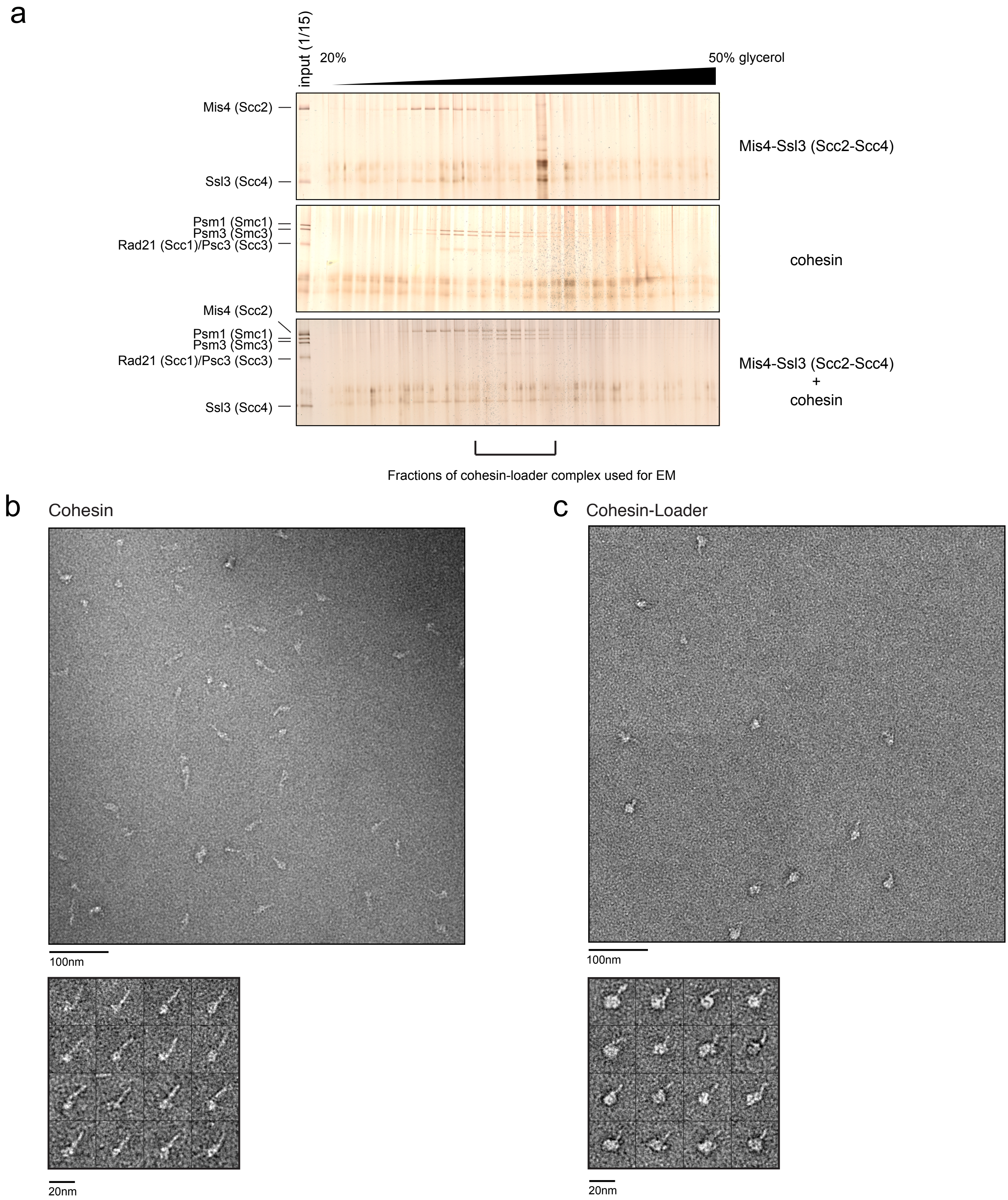

Supplementary Figure 5. EM analysis of cohesin and cohesin-loader complex

(a) Silver staining of fractions from glycerol-gradient centrifugation showing the formation of the S. pombe cohesin-loader complex.

(b) Top panel: representative micrographs of cohesin under negative-stain EM. Bottom panel: selected images of cohesin. The molecules have a “rod-shape” conformation with a head likely composed of the Smc ATPase head domains, Scc1, and Scc3, and an extended tail composed of Smc coiled coils.

(c) Top panel: representative micrographs of cohesin-loader complex under negative-stain EM. Bottom panel: selected images of cohesin-loader complex. The molecules also have a “rod-shape” conformation with a head likely composed of the Smc ATPase head domains, Scc1, Scc3, Scc2-Scc4, and an extended tail composed of Smc coiled coils.

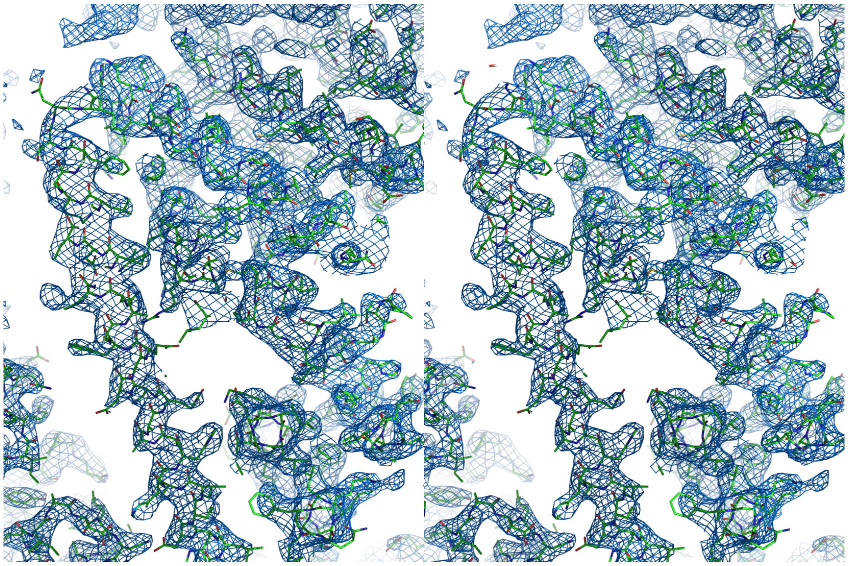

Supplementary Figure 6. Stereo image of a portion of the electron density map.
Section of final 2|Fo|-|Fc| map contoured at 1σ.
